# High Resolution Intravital Imaging of the Renal Immune Response to Injury and Infection in Mice

**DOI:** 10.3389/fimmu.2019.02744

**Published:** 2019-11-29

**Authors:** John Sedin, Antoine Giraud, Svava E. Steiner, David Ahl, A. Erik G. Persson, Keira Melican, Agneta Richter-Dahlfors, Mia Phillipson

**Affiliations:** ^1^Department of Medical Cell Biology, Uppsala University, Uppsala, Sweden; ^2^Swedish Medical Nanoscience Center, Department of Neuroscience, Karolinska Institutet, Stockholm, Sweden

**Keywords:** intravital, renal infection, neutrophils, macrophages, mononuclear phagocytes, sterile injury

## Abstract

We developed an experimental set up that enables longitudinal studies of immune cell behavior *in situ* in the challenged as well as unchallenged kidney of anesthetized mice over several hours. Using highly controlled vacuum to stabilize the kidney, the superficial renal cortex could continuously be visualized with minimal disruption of the local microenvironment. No visible changes in blood flow or neutrophils and macrophages numbers were observed after several hours of visualizing the unchallenged kidney, indicating a stable tissue preparation without apparent tissue damage. Applying this set up to monocyte/macrophage (CX_3_CR1^GFP/+^) reporter mice, we observed the extensive network of stellate-shaped CX_3_CR1 positive cells (previously identified as renal mononuclear phagocytes). The extended dendrites of the CX_3_CR1 positive cells were found to bridge multiple capillaries and tubules and were constantly moving. Light induced sterile tissue injury resulted in rapid neutrophil accumulation to the site of injury. Similarly, microinfusion of uropathogenic *Escherichia coli* into a single nephron induced a rapid and massive recruitment of neutrophils to the site of infection, in addition to active bacterial clearance by neutrophils. In contrast, the kidney resident mononuclear phagocytes were observed to not increase in numbers or migrate toward the site of injury or infection. In conclusion, this model allows for longitudinal imaging of responses to localized kidney challenges in the mouse.

## Introduction

The host immune response to injury and infections is both site- and situation-dependent, and is even influenced by the circadian rhythm, with variations in the mechanisms of leukocyte recruitment to different organs as well as to different infectious agents (1–3). In general, tissue resident immune cells acting as sentinels, rapidly detect changes of the microenvironment, and respond accordingly. This response includes macrophage phagocytosis and killing of the pathogens, but also production of chemokines and cytokines to activate and recruit other immune cells to the site. The first cells to be recruited from circulation to the site of infections are neutrophils, which are competent bacteria-killers via the production of reactive oxygen species and antibacterial products. In addition to their role in host defense, leukocytes contribution to tissue restoration following injury has recently been recognized (4–6).

In contrast to the urethra and the bladder, the kidney is typically considered sterile (7). However, urinary tract infections (UTI) are one of the most frequently reported bacterial infections, both in community and hospital settings (8). In some cases, the infection can spread from the urethra to the bladder and eventually further up into the kidney and, in the worst scenario, enter the blood circulation to cause urosepsis. During homeostasis, the urinary tract eliminates these microorganisms rapidly and efficiently by natural host defense mechanisms including physical (washing out, secretory IgA antibodies), chemical (pH, antibacterial peptides), and biological (immune cells) weapons (9). The healthy kidney contains at least five distinct populations of resident renal mononuclear phagocytes with overlapping dendritic and macrophage characteristics (10–12). These cells appear homogenously distributed throughout the kidney (13) and are derived either from the yolk sac during embryogenesis, or from hematopoietic stem cells by *de novo* recruitment of circulating monocytes (14). Activation of the resident renal mononuclear phagocytes is associated with the development and progression of kidney disease (15). However, the precis role of these numerous cells in maintaining and regulating kidney functions is not yet fully clarified. The sparse information regarding the function of resident renal mononuclear phagocytes can be partially attributed to the difficulties in studying them locally *in vivo* rather than as a whole population in an organ. Due to the high tissue density of the kidney, intravital imaging is limited to the superficial regions of the cortex and to the use of fluorescence, either from conjugated antibodies directed against specific surface markers or from genetically labeled cell populations which endogenously express fluorophores. Further, the correlation between levels of antibody-targeted surface molecules/fluorescently tagged gene and the phenotype of the cells needs to be confirmed. Overlapping expression of classical markers for dendritic cells and macrophages further contribute to confusion within the field regarding the action of these tissue resident populations.

Previously, we had developed a spatially-temporally controlled model of kidney infection in rats which allowed us to visually follow the progression of uropathogenic *Escherichia coli* (UPEC) kidney infection in real-time (16, 17). UPEC express a unique sub-set of virulence factors which allow them to colonize the urinary tract including adhesion pili and exotoxins (18). In this model, bacteria are microinfused into a single tubule of an exposed kidney in an anesthetized rat and followed with multiphoton microscopy. This work was one of the first to use intravital imaging to follow bacterial infection *in situ* and described a number of new phenomena including a rapid protective vascular coagulation response (17) and the role of bacterial virulence factors in host response kinetics (19). An aim had been to follow up on this work by detailing the early immune cell recruitment to UPEC infection, but a drawback in this model was the lack of tools available for rats, particularly a lack of fluorescently transgenic animals as are available in mouse models. In this current report, we describe an experimental set up that enables studies of immune cell behavior in the cortex of the mouse kidney, including transgenic animals, in both healthy animals and during injury and local bacterial infection in single nephron over several hours. Using this set up, the extended dendrites of the resident renal mononuclear phagocytes were found to bridge multiple capillaries and tubules and were in constant motion, apparently probing the environment of the healthy kidney. In addition, intravascular neutrophils were demonstrated to crawl within the microvasculature. Following both sterile injury and infection, the neutrophils rapidly accumulated in large numbers at the afflicted site, where they also were found to phagocytose bacteria. Neither sterile tissue injury nor bacterial infection however, did alter the mononuclear phagocytes network, which did not increase in number or migrated following the insult. In summary, this study demonstrates that a stable preparation for intravital microscopy of the mouse kidney cortex reveals distinct immune cell behavior *in situ* and can help to better understand their role and interactions.

## Materials and Methods

### Animals

C57Bl/6J mice [30–35 g (Taconic, Denmark)], CX_3_CR1^+/GFP^ F1 hybrid mice [25–30 g (B6.129P-Cx3cr1tm1Litt/J, The Jackson Laboratory, (20)) and (C57Bl/6J, Taconic)] and CX_3_CR1^+/CRE^: Rosa-Tomato hybrid mice [25–30g (Cx3cr1^tm2.1(cre/ERT2)Jung^ (21)) and Gt(ROSA)26Sor^tm14(CAG−tdTomato)Hze^ (22)] were used. All animals had access to tap water and pelleted food *ad libitum* throughout the experimental study. The following antibodies were administered intravenously to visualize kidney resident mononuclear phagocytes, blood vessels, platelets and neutrophils, respectively: anti-mouse; F4/80, CD31 (PECAM-1), CD41, and Ly-6G (Gr-1). A detailed information about clone, suppliers, doses and conjugated fluorophores are presented in the Supplementary Table 1. The antibodies were administered approximately 30 min before start of experiments to optimize the staining of the target cells. All experiments were approved by the Regional Animal Ethics Committee in Uppsala, Sweden, under the ethical permit number C98/16.

### Surgical Preparation, Tissue Stabilization, and Confocal Imaging (Figure 1A)

**Figure 1:**
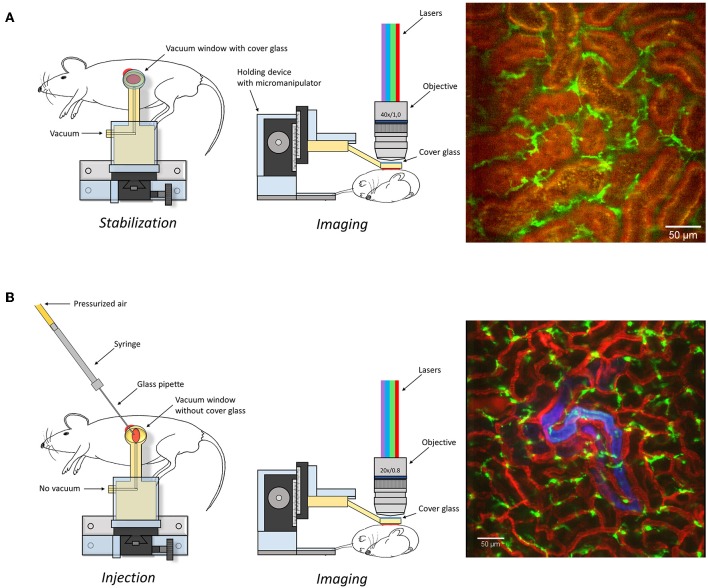
Graphical depiction of the experimental set-up described in the manuscript to expose **(A)** or microinfuse **(B)** and image the kidney. An image captured under these settings is presented as an example on right of the graphical description. Scale bar represents 50 μm. Additional information is available in Supplementary Figure 1 and Supplementary Video 1.

Mice were anesthetized by spontaneous inhalation of isoflurane (Abbott Scandinavia, Sweden) diluted 1.8–2.6% in a mixture of air and oxygen. The animals were immediately placed on a custom made table with a heating pad to control and maintain body temperature. The left jugular vein was catheterized with a PE-10 cannula for the injection of antibodies and saline (detailed information in Supplementary Table 1). Mice were then placed in the right, lateral decubitus position and a small incision into the left side of the abdominal cavity was made to expose the left kidney. To allow for real time imaging of the live kidney *in situ*, we constructed a modified version of the pancreatic vacuum window (23) with a 4 × 6 mm internal diameter. The window was covered with an imaging-grade 13 mm coverslip held in place with vacuum grease. The suction window (Supplementary Figure 1), attached to an in house constructed stand, was guided in position immediately above the kidney (Figure 1A). A low vacuum (8–16 mbar) was applied before lowering the window in contact with the tissue. In each experiment the lowest vacuum needed for immobilization was used to prevent kidney damage. The table was then transferred to a line-scanning confocal microscope (Zeiss LSM 5 Live with Zeiss Zen 2009 software) and the objective (WPlanApo 40x/1.0 objective with 0.5x optical zoom) was lowered into the central region of the preparation to minimize any effects of suction transmitted to the edges of the tissue. A graphical description and pictures of the experimental setup are presented in Figure 1A and Supplementary Figure 1. For confocal fluorescence microscopy we used the Zeiss LSM 5 Live line-scanning microscope with simultaneous two-channel acquisition. This system is equipped with maintenance-free lasers of diode or solid-state type, 405 nm laser diode, 50 mW; 488 nm laser diode, 100 mW; diode-pumped solid-state laser 532 nm, 75 mW; laser diode 635 nm. Excitation filters, BP 415–480 for BV421 and eFluor450; BP 500–525 for GFP and Alexa 488; BP 550–615 or LP 505 for TRITC, BV605 and Alexa 555; LP 650 for BV650, Alexa 647 and eFluor660 combined with suitable beam splitters and lasers. Brightfield intravital microscopy was done using a with a X-Cite 120 PC fluorescence system (EXFO Photonic Solutions Inc, Canada).

### Light Induced Tissue Injuries

Large injuries were induced by exposing the kidney to X-Cite 120 PC fluorescence system (EXFO Photonic Solutions Inc, Canada) at high intensity. Exposing the kidney to a short (10 s) burst of high intensity (50 mW) of the 405 nm excitation laser of the line-scanning confocal microscope induced a small scope in the renal cortex.

### Bacterial Infection

The UPEC strain LT004 (16) which constitutively expresses GFP from a chromosomal insertion was used in infection experiments. Microinfusion was performed by adapting to mouse the method previously described for rat (16, 17, 24) Briefly, inoculum were grown overnight in Luria Broth (LB) with chloramphenicol (20 mg/μl) at 37°C. A 1:100 dilution of the overnight culture was then reinoculated into LB with chloramphenicol (20 mg/μl) and grown at 37°C to an OD600 of 0.6. The bacteria were then washed twice (5 min 5 kg) in NaCl (154 mM) and suspended in PBS to a final concentration of 1 × 10^9^ CFU/ml. The final bacterial suspension used to microinfuse the kidney tubules were made by mixing the bacterial stock with the injection tracer solution in a 1:1 ratio, giving a final bacterial solution of 5 × 10^8^ cfu ml^−1^. The tracer solution was a mixture of 0.7 mg ml^−1^ Fast Green FCF (Fisher, Fair Lawn, NJ, USA) and 0.4 mg ml^−1^ Alexa Fluor 647 conjugated 10 kDa dextran.

#### Bacteria Infusion Into the Renal Tubule

Needles for the microinfusion were made from thin walled borosilicate glass capillaries of 1 mm outer diameter containing an inner filament (WPI TW100F-4) by pulling the capillary in opposite directions with an in house constructed micropipette puller. The tip of the needle was cut off at the desired tip diameter of 8–10 μm using a Dumont 5/45 forceps and sanded to a 30° tip angle. Before using the micropipette, the pipette was function tested by injecting air in 1 M hydrochloric acid, injection pressure 20 psi. After the test the tip was gently dried with a soft tissue.

#### Pipette Filling

The capillary was mounted to the nozzle of a pneumatic PicoPump (WPI PV820) on the hold pressure port with the vent port connected to an industrial vacuum line. Using a Leitz micromanipulator and holder to hold the pipette the tip was lowered into the bacterial suspension and filled from the tip using vacuum pressure.

#### Microinfusion (Figure 1B)

The capillary was mounted to the nozzle of the PV820 on the eject pressure port with the vent port connected to the atmosphere. Using the hold pressure gauge the hold pressure was set to 2 psi. Under stereoscopic microscope observation (96x), using a Leitz micromanipulator and pipette holder, the bacterial suspension was injected for ~10 min into one renal tubule in mouse, prepared as described for confocal imaging (described above). The rate of infusion averaged 49 ± 23 nl min-1 (*n* = 7), which corresponded to delivery of about 5 × 10^5^ cfu. In sham-infected animals sterile PBS mixed with tracer solution was infused into the renal tubules. Observation was performed with a PlanApo 20x/0.8 objective with 0.6 up to 0.8x optical zoom in the confocal microscope described above.

## Results

### The Experimental Setup Allows Maintained Kidney Integrity

**Figure 2:**
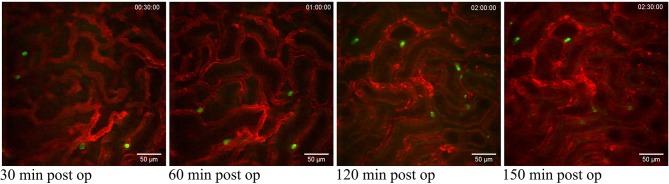
Neutrophils scanning capillaries within the superficial cortex in the unchallenged kidney. Confocal snapshots of the unchallenged kidney in a live C57BL/6J mouse taken at 30, 60, 120, and 150 min post-operation from the Supplementary Video 3. Capillaries are visualized in red (anti-CD31- mAb Alexa Fluor 555) and circulating neutrophils in green (anti-Ly6G-mAb Alexa Fluor 488). Scale bars are 50 μm and pictures are representative of 5 experiments. WPlanApo 40x/1.0 objective with 0.5 zoom was used.

The superficial renal cortex was continuously visualized for several hours in anesthetized mice under an upright laser-scanning confocal microscope using the experimental setup described in Figure 1A and Supplementary Figure 1. The kidney was stabilized within the peritoneal cavity by means of an in-house constructed vacuum stabilized observation window (23) to avoid the use of a kidney cup that can alter circulation, accelerate dehydration, and drop in organ temperature. Further, the use of an upright microscope facilitate long *in vivo* observations with maintained peripheral circulation, as it allows for the organ of interest to be situated at the level of the heart to avoid development of edema. To minimize the disruption of renal tissue integrity and microcirculation by the stabilizing holding arm, the size of the window was enlarged compared to the original design (23) to spread the low, negative gas pressure over a larger surface area (Supplementary Figure 1). Using this approach, auto-fluorescent proximal tubule and CD31 stained blood vessels with visible blood flow could be detected over time to a depth up to 100 μm (Supplementary Video 1). No visible major changes in blood flow were detected after 5 h post operation (Supplementary Video 2). Administration of anti-mouse Ly-6G antibodies intravenously revealed the presence of neutrophils interacting with capillary endothelium (Figure 2 and Supplementary Video 3), demonstrating that in our setting neutrophils normally scan the vasculature of the renal cortex in the unchallenged kidney. Repeated imaging over a period of more than 2 h of the intact live kidney did not show any clear increase in the number of visible detected neutrophils (Figure 2) or the number of adherent and/or crawling neutrophils. Thus, this experimental setup allows for visualization of the kidney cortex in the anesthetized mouse without concomitant obvious or dramatic neutrophil activation and recruitment hours after exposure.

### Renal Network of Probing Mononuclear Phagocytes

**Figure 3:**
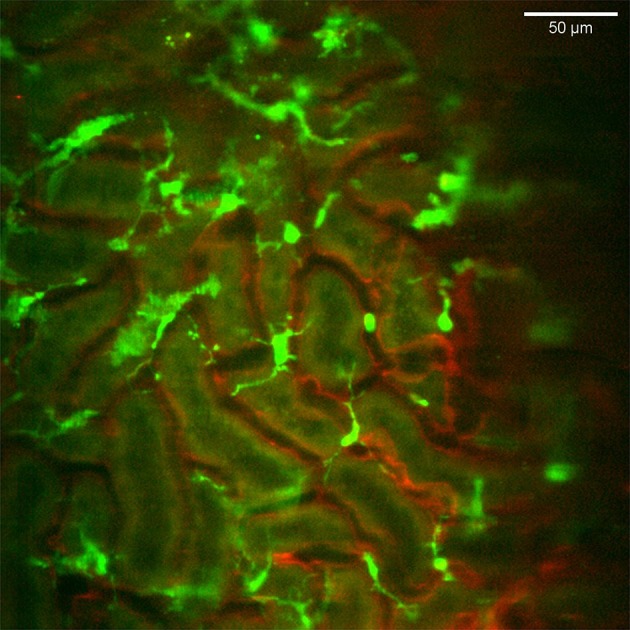
Distribution and stellar shape of CX_3_CR1 positive mononuclear phagocytes in the renal cortex. Confocal snapshot from the Supplementary Video 4. Blood vessels in red (eFluor 450 conjugated anti-CD31 mAb) and mononuclear phagocytes in green (CX_3_CR1-GFP). The scale bar indicates 50 μm. Recorded through a WPlanApo 40x/1.0 objective with 0.5 zoom. Additional 3 dimensional observations are presented in Supplementary Video 5.

**Figure 4:**
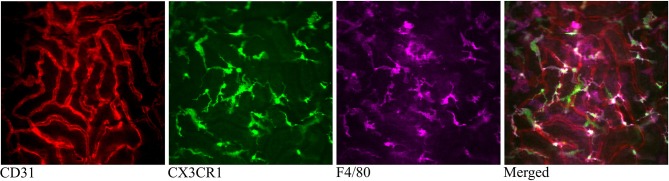
Confocal snapshot of the live kidney in a CX_3_CR1^+/GFP^ mouse taken 30 min after intravenously administered anti-F4/80-mAb-Alexa Fluor 647. Capillaries are displayed in red (anti- CD31-mAb eFluor 450), mononuclear phagocytes in green (CX_3_CR1-GFP) and in magenta (anti-F4/80- mAb Alexa Fluor 647). Double positive cells appear bright in the merged figure. All scale bars represent 50 μm. WPlanApo 40 x/1.0 objective with 0.5 zoom was used.

**Figure 5:**
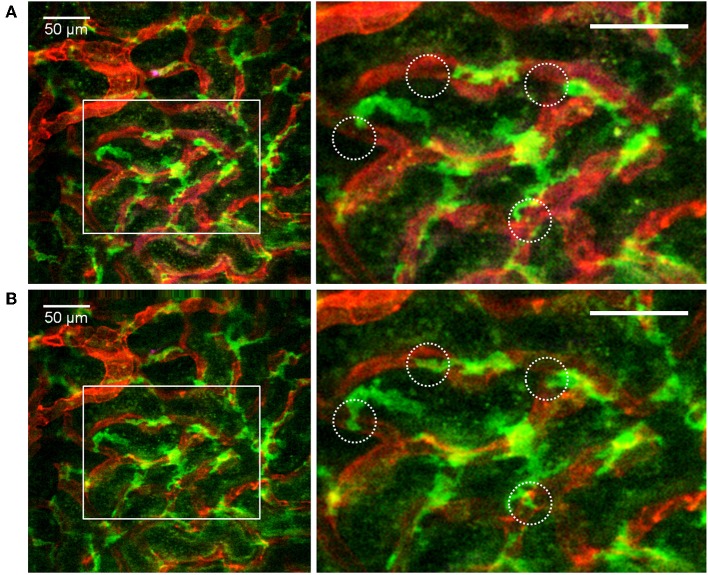
Distribution and dendritic activity of F4/80^+^ mononuclear phagocytes in the renal cortex. Confocal snapshot at different time points from the Supplementary Video 6. **(A,B)** Show of perivascular F4/80^+^ mononuclear phagocytes sampling the renal capillaries. Capillaries are displayed in red (anti-CD31-mAb Alexa Fluor 555) and mononuclear phagocytes in green (anti-F4/80-mAb Alexa Fluor 647). Boxed areas highlight several active cells with extension and retraction of dendrites (circles). Scale bar 50 μm. WPlanApo 40x/1.0 objective with 0.5 zoom was used.

The CX_3_CR1 chemokine receptor is a commonly used marker for macrophages, monocytes and some dendritic cells. Using a transgenic mouse strain expressing the green fluorescent protein under the regulatory signal governing the expression of CX_3_CR1 (20) we monitored the distribution and behavior of these cells in the kidney. Confocal microscopy of CX_3_CR1^+/GFP^ mouse kidney *in situ* in anesthetized mice revealed an extensive network of stellate-shaped CX_3_CR1 positive cells in the interstitium of the superficial renal cortex (Figure 3 and Supplementary Video 4). As previously described, most of these cells, also called renal mononuclear phagocytes (rMoPhs) (25), demonstrated transcapillary and transtubular connections with their multiple dendrites bridging several tubules and capillaries (Figure 3 and Supplementary Videos 4, 5) (13, 26). Further, *in vivo* visualization verified that the majority of the CX_3_CR1 positive cells located in the cortex were also positive for the pan-macrophage marker F4/80 (Figure 4), confirming previous observations (13, 27). These were situated in close proximity to the tubular capillaries (Figure 3 and Supplementary Videos 4, 5). Time laps recording experiments over 1 h showed dendritic activity of these cells, and they seemed to be constantly probing and sampling the vasculature as well as the tubules (Figure 5 and Supplementary Video 6). These observations confirm previously reported observations by other groups and further validate that our experimental does not seem to alter the rMoPhs network (13, 26).

### Recruitment of Neutrophils to Renal Sterile Injury or Bacterial Infections

**Figure 6:**
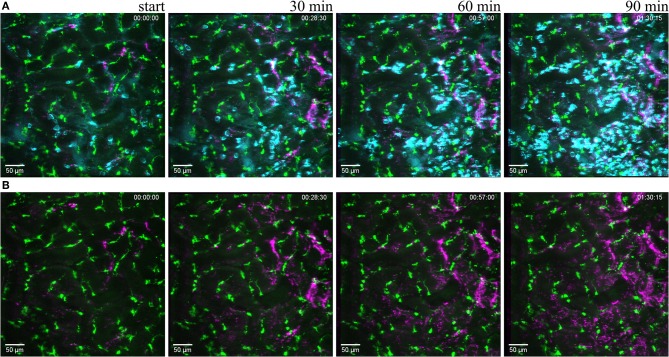
Reaction to light induced sterile injury. Snapshot at 0, 30, 60, and 90 min of Supplementary Video 7 from CX_3_CR1^+/GFP^ mouse kidney after over exposure under a fluorescence lamp at maximum intensity to induce large area damage. Recorded through a PlanApo 20x/0.8 objective with 0.8 zoom. Scale bar 50 μm. (**A** related to Supplementary Video 7) Mononuclear phagocytes displayed in green (CR_3_CR1-GFP), neutrophils in cyan (anti-Ly6G-mAb Brilliant Violet 421) and platelets in magenta (anti CD41-mAb Brilliant Violet 605) to visualize blood flow. (**B** related to Supplementary Video 8) Same picture as in **(A)** without the neutrophil fluorescence for a better observation of the CR_3_CR1-GFP cells and platelet accumulation.

To explore if our experimental setting did not altered the immune reactivity of the exposed mouse kidney, we tried to visualize the recruitment of circulating neutrophils to injury site. A superficial injury on the kidney was induced by over exposing it to intense light sources from a fluorescence illumination system or a high intensity 405 nm laser that, respectively, can induce a large or a small burn injury in the cortex. The light induced damages led to a rapid and massive accumulation of neutrophils to damaged area (Figure 6A and Supplementary Video 7). In the case of smaller local laser-induced damage, neutrophils recruited from circulation were also detected in close proximity of the injury (Supplementary Figure 2). The large network of rMoPhs was not affected by the sterile injuries. Indeed no obvious changes were observed in the pattern of the CX_3_CR1 positive cell network at the site of neutrophil accumulation (Figure 6B and Supplementary Video 8). This observation corroborates the description of CX_3_CR1 positive cells acting merely as sentinels activated upon injury to release chemokines that rapidly trigger the recruitment of neutrophils at the early stage of bacterial infections (15, 28).

**Figure 7:**
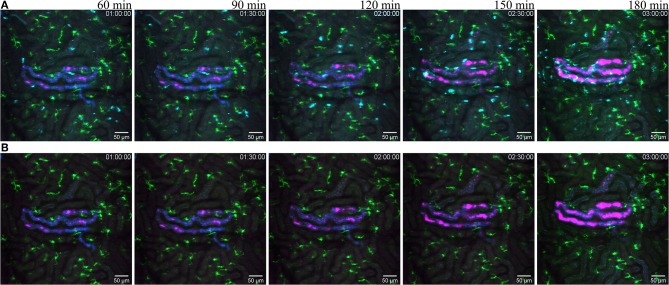
Reaction to infected tubule. (**A** related to Supplementary Video 9) snapshot taken at 60, 90, 120, 150, and 180 min post infection of one tubule with the UPEC strain (GFP displayed in magenta) in a of a CX_3_CR1^+/CRE^:Rosa-Tomato mouse recorded through a PlanApo 20x/0.8 objective with 0.7 zoom. The injected bacterial solution contains an injection tracer (blue, Alexa Fluor 647 conjugated dextran) to visualize injected tubule. Neutrophils are shown in cyan (anti-Ly6G mAb Brilliant Violet 421). CX_3_CR1 positive cells are displayed in green (tdTomato). (**B** related to Supplementary Video 10) Same picture as in **(A)** without the neutrophil fluorescence for a better observation of the CR_3_CR1-GFP cells and bacterial growth.

**Figure 8:**
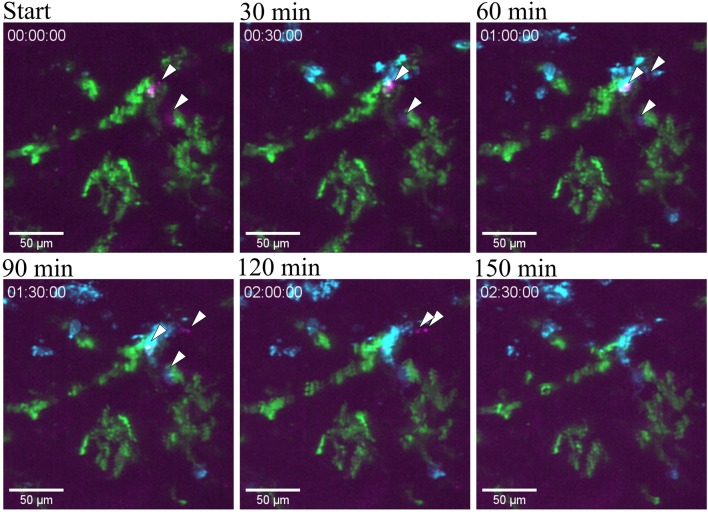
Bacterial clearance. Snapshots taken from Supplementary Video 10 at different time point post infection of one tubule infused with the UPEC strain (GFP) in a of a CX_3_CR1^+/CRE^:Rosa-Tomato mouse showing the clearing of bacteria by neutrophil in red (Ly6G mAb-BV421). CX_3_CR1 positive cells are displayed in green (tdTomato) and UPEC in magenta (GFP). PlanApo 20x/0.8 objective with 0.6 zoom.

To observe the immune cell reaction induced by an infectious stimulus, we injected 10^5^ cfu of a fluorescent uropathogenic *Escherichia coli* strain (UPEC) directly into a single renal tubule adapting a method developed in the rat to the mouse [Figure 1B, (16)]. Observation of the infected site over several hours revealed the dynamics of bacterial growth and tubule colonization as well as neutrophils moving to the infected site (Figure 7A and Supplementary Video 9). The presented experimental setting do thus maintain the abilities to detect and signal a local infection that lead to neutrophil chemotaxis toward to infected regions in the kidney cortex. While neutrophils were observed close to the infected site at the late time points, the CX_3_CR1 positive cells network appeared similar compared to prior to when the bacteria were injected (Figure 7B and Supplementary Video 10). This observed relative passivity of macrophages compared to neutrophils in phagocytosis during infection in the upper urinary tract agrees with results present in the literature (29). Interestingly sham-infusion of PBS alone in a tubule did not induce neutrophil recruitment, implicating the bacteria as the attracting inflammatory agent and showing that tubuli puncture *per se* was not sufficient to trigger a massive neutrophil response. Moreover, the setting allowed for real time visualization of bacterial clearance by neutrophils as we could observe that the GFP signal originating from infecting bacteria decreased and finally disappeared in parallel to neutrophils becoming GFP-positive (Figure 8 and Supplementary Video 11).

The correlating rat model of UPEC infection has previously shown that upon UPEC infection in a single renal tubule, coagulation is triggered in the local peri-tubular capillaries (17, 30). This coagulatory response observed several hours after infection has been shown to be protective by isolating the infection site and preventing bacterial translocation into the blood stream and a progression to urosepsis (17). In the mouse setting, some experiments were carried out with circulation tracers. These reporters allowed monitoring the quality of blood flow during observation. Light injury induced important perturbation in the circulation, as reveled by the accumulation of platelets in the capillaries surrounding the damaged area (Figure 6B and Supplementary Video 7). Tubule infusion in the mouse is a delicate manipulation to perform. Going through the kidney capsule can be difficult and can lead to either perforation of capillaries or bacterial leakage. Only few infusions had been successfully performed and the very limited number of observations could neither allow us to conclude if this clotting around infected tubules is or isn't also a protective mechanism in the mouse. However, trials that resulted in bacterial leakage or local bleeding, both with UPEC or PBS, lead to an observable perturbation in blood flow quality in the capillaries as the circulation tracer was not displaced by passing erythrocytes as revealed by capillaries segments emitting intense fluorescent signal from the circulation tracer (Supplementary Video 12). The presented model may thus be used to investigate local coagulation in capillaries surrounding an infected tubule as has been done in the rat model (17).

## Discussion

This study demonstrates means to longitudinally study the behavior of resident and recruited immune cells in kidney cortex under basal conditions as well as during localized tissue injury and infection *in vivo* in the mouse. This model offers many genetic tools to visualize and decipher immune cell behavior and interaction by direct visualization. Using this approach, we could observe the numerous stellate-shaped CX_3_CR1^+^ and F4/80^+^ resident renal mononuclear phagocytes, which were found scattered throughout the kidney, where they exerted a probing behavior as their extended dendrites spanned several tubules and capillaries, and were in motion. Further, neutrophil scanning of the kidney vasculature was observed under basal conditions, and massive neutrophil accumulation was detected following light-induced sterile injury or bacterial infection, whereas the structure of the renal mononuclear phagocytes network was not affected by either challenge.

The development of experimental models to study biological events in the living animal is both difficult and time consuming. Great care must be taken no to disturb the function and integrity of the organ studied. As the kidney is a very delicate organ that is highly dependent on an intact blood flow, it is a demanding organ to study *in vivo*. By carefully applying a gentle controlled negative pressure (vacuum) to the outer surface of the kidney, the vacuum suction window provided both stabilization and optimal tissue preparation for imaging. Using bright and photostable fluorophores, e.g., Alexa Fluor dyes, the risk of phototoxicity was minimized. Further, a multi-photon microscope would improve the z-resolution and further reducing the risk of phototoxicity, making it possible to image even deeper into the tissue. Importantly, this method enables imaging *in situ* and does not involve removal of the organ from the animal, allowing for the study of infection with the vasculature, nervous, and immune systems intact. Further, with this method we were able to image beyond 5 h post-op, which is probably close to the time limit a mouse can be kept anesthetized under our experimental conditions without severely affecting the blood pressure.

Animal models of inflammation has together with intravital microscopy been instrumental for increasing the understanding of the mechanisms underlying the leukocyte recruitment from circulation to tissue, as well as leukocyte effector function at different inflamed sites. While the high density of renal tissue impedes traditional bright field imaging of its vasculature and immune cells, high-speed confocal microscopy of fluorescently marked cells, and/or structures enables visualization of blood flow and cell-cell interactions to limited depth in living animals. Intravital microscopy is inevitable associated with both circulatory and respiratory movements, which highlights the importance of reliable models where these cells can be visualized during minimal organ stress. By utilizing a modified version of the technique presented by Looney et al. (23), we have established a method for real-time analysis of the renal micro-environment which is a powerful tool to learn more about the renal resident immune cells during homeostasis as well as during the pathology of bacterial infections *in vivo*. The present technique provides access to the intact microcirculation and microanatomy of the mouse kidney, with the combination of easy to use, high resolution, great stability and with maintenance of normal immune cell reactivity.

In the healthy kidney, numerous mononuclear phagocytes are scattered throughout the renal tissue. The mononuclear phagocytes can be divided into several subpopulations dependent on their expression levels of macrophage- and dendritic cell markers (F4/80, CD11b, and CD11c, respectively). Recent gene array data combined with antibodies directed against CD64 and MerTK (efferocytosis receptor) indicate that the majority of these cells are macrophages, and not dendritic cells as previously believed (11, 31). In addition to their distinct identities, their diverse effector functions remains to be fully established. Clodronate-depletion removes actively phagocytosing cells and resulted in lower plasma creatinine levels following kidney injury, demonstrating that these macrophages aggravate acute kidney injury (32). However, when the renal mononuclear phagocytes are depleted to a larger extent using the CD11b-diphtera toxin receptor model, protection against ischemia-reperfusion injuries is not observed (33), which indicates that some of the renal mononuclear phagocytes have important functions in tissue recovery.

The role of both neutrophils and renal mononuclear phagocytes in bacterial infections has previously been described (9, 15, 29). Using a model of repeated transurethral installation of *E. coli* (UPEC) into the bladder, flow cytometry following kidney homogenization demonstrated that renal neutrophil- but not macrophage numbers were increased 3 h following bacterial instillation, while the total number of dendritic cells and macrophages decreased over time (29). Further, the mononuclear phagocytes were shown to upregulate CXCL2 in response to infection, and thereby contributed to the recruitment of neutrophils. The method developed in the current manuscript enabled tracking of local immune cell behavior over time in the renal cortex of unaffected kidneys or following sterile injury or bacterial infections. We observed that neutrophils of the healthy kidney displayed a normal behavior, as they were sporadically scanning the inside of the blood vessels, and, in response to injury and infection, rapidly accumulate in large numbers at the affected site. The renal mononuclear phagocytes of healthy kidneys were as expected actively scanning their local microenvironment with their dendrites spanning several tubules and capillaries, but, in contrast to neutrophils, did not migrate toward adjacent sterile injury or bacterial infection within the observation time. This is in contrast to what is described for the sterile injury of the liver (34), where F4/80^+^ cells originating from the peritoneal cavity accumulated as early as 1 h following insult. Cells of the innate immune system are classically viewed as first responders to invasion and damage, where they contribute in different ways to restore tissue homeostasis. It is clear that their ability to detect and respond to environmental signals differ between challenges as well as affected sites (3, 35).

In summary, we established a model that enables longitudinal and precise imaging of the superficial kidney cortex for hours in mouse without impacting the circulation and immune cell potential. This model allowed us to visualize the behavior of neutrophils and resident mononuclear phagocytes submitted to localize aseptic as well as septic challenges.

## Data Availability Statement

All datasets generated for this study are included in the article/Supplementary Material.

## Ethics Statement

The animal study was reviewed and approved by Regional Animal Ethics Committee in Uppsala, Sweden.

## Author Contributions

JS, AG, and DA conducted all the experiments. JS, AG, SS, KM, DA, AP, ARD, and MP designed the study, analyzed the data, interpreted the results. JS, AG, KM, and MP wrote the manuscript. All authors contributed to the editing of the manuscript, read and approved the final version before submission.

### Conflict of Interest

The authors declare that the research was conducted in the absence of any commercial or financial relationships that could be construed as a potential conflict of interest.

## Supplementary Material

Supplementary Video 1 related to Figure 1A 3D projection of a z-stack taken with a WPlanApo 40x/1.0 objective with 0.5 zoom showing autofluorescent tubules in green and labeled blood vessels in red (Alexa Fluor 555 conjugated anti-CD31 mAb). The stack (depth 83 μm) was exported to video at 12 fps using ImageJ.

Supplementary Video 2 Visualization of blood flow. Live imaging of the kidney of a CX_3_CR1^+/GFP^ mouse 5 h after surgery taken with a WPlanApo 40x/1.0 objective with 0.5 zoom. Blood flow was imaged by i.v. injection of TRITC dextran 500 kDa as contrast agent that allow the observation of erythrocytes as black (not stained) dots. Sequence was recorded at a rate of 2 fps for 150 s and then exported to video at 12 fps using ImageJ. GFP fluorescence from the CX_3_CR1 positive cells not displayed.

Supplementary Video 3 related to Figure 2 Neutrophils scanning capillaries within the superficial cortex in the unchallenged kidney of a C57Bl/6J mouse with a WPlanApo 40x/1.0 objective with 0.5 zoom. Blood vessels are labeled with Alexa Fluor 555 conjugated anti-CD31 mAb (red) and neutrophils are seen in green (Alexa Fluor 488 conjugated anti-Ly6G mAb). Elapsed time is shown at the low right. The time lapse was recorded directly after surgery at 0.3 fps and exported to video at 24 fps using ImageJ. The scale bar indicates 50 μm.

Supplementary Videos 4, 5 related to Figure 3 Video 4 Distribution of CX_3_CR1 positive mononuclear phagocytes in the renal cortex of CX_3_CR1^+/GFP^ mouse. Blood vessels are in red (eFluor 450 conjugated anti-CD31 mAb) and mononuclear phagocytes in green (CX_3_CR1-GFP). Elapsed time is shown at the low right. The time lapse was recorded at 6 fps with a WPlanApo 40x/1.0 objective with 0.5 zoom and exported to video at 24 fps using ImageJ. The scale bar indicates 50 μm. Video 5: 3D projection of a z-stack (depth 86.74 μm) taken on the same mouse with a WPlanApo 40x/1.0 objective with 0.5 zoom showing blood vessels in red (eFluor 450 conjugated anti-CD31 mAb), CX_3_CR1 positive cells in green (GFP) and neutrophils in cyan (Alexa Fluor 555 conjugated anti-Ly6G mAb).

Supplementary Video 6 related to Figure 5 Distribution and dendritic activity of F4/80^+^ mononuclear phagocytes in the renal cortex of a C57Bl/6J mouse. Blood vessels are displayed in red (Alexa Fluor 555 conjugated anti-CD31 mAb) and mononuclear phagocytes in green (AF647 anti-F4/80 mAb) and neutrophils in magenta (Alexa Fluor 488 conjugated anti-Ly6G mAb). Elapsed time is shown at the low right. The time lapse was recorded at 0.3 fps with a WPlanApo 40x/1.0 objective with 0.5 zoom and exported to video at 24 fps using ImageJ. The scale bar indicates 50 μm.

Supplemental Video 7 related to Figure 6A Recruitment of neutrophils to light induced sterile injury. The CX_3_CR1^+/GFP^ mouse kidney following exposure under a fluorescence lamp at maximum intensity to induce a large damaged area. Mononuclear phagocytes are displayed in green (GFP), neutrophils in cyan (Brilliant Violett 421 conjugated anti-Ly6G-mAb) and platelets in magenta (Brilliant Violett 605 conjugated anti CD41) to visualize blood flow. Elapsed time is shown at the low right. Twenty stacks (depth: 51.7 μm) were recorded with 5 min intervals with a PlanApo 20x/0.8 objective with 0.8 zoom and exported to video at 9 fps using ImageJ (3D-Projection). The scale bar indicates 50 μm.

Supplementary Video 8 related to Figure 6B Light-induced sterile injury, same video as Supplementary Video 7 related to Figure 5A without the neutrophil fluorescence to better visualize the behavior of CX_3_CR1 positive cells and blood flow. The CX_3_CR1^+/GFP^ mouse kidney following exposure under a fluorescence lamp at maximum intensity to induce a large damaged area. Mononuclear phagocytes are displayed in green (GFP) and platelets in magenta (Brilliant Violett 605 conjugated anti CD41) to visualize blood flow. Elapsed time is shown at the low right. Twenty stacks were recorded with 5 min intervals with a PlanApo 20x/0.8 objective with 0.8 zoom and exported to video at 9 fps using ImageJ. The scale bar indicates 50 μm.

Supplementary Video 9 related to Figure 7A Recruitment of neutrophils to an infected tubule. Live imaging of the kidney area surrounding one tubule infected with the UPEC strain from 90 to 155 min post infection. Bacteria are displayed in magenta (GFP), mononuclear phagocytes in green (tdTomato) and neutrophils in cyan (Brilliant Violett 421 conjugated anti-Ly6G mAb). Elapsed time is shown at the upper right. Fourteen stacks (depth: 53.5 μm) were recorded with 5 min intervals with a PlanApo 20x/0.8 objective with 0.7 zoom and exported to video at 9 fps using ImageJ (3D-Projection). The scale bar indicates 50 μm.

Supplementary Video 10 related to Figure 7B Live imaging of the kidney area surrounding one tubule infected with the UPEC strain from 90 to 155 min post infection. This is the same video as Supplemental Video 9 related to Figure 7A without the neutrophil fluorescence to better visualize the behavior of CX_3_CR1 positive cells around the infected tubule. Bacteria are displayed in magenta (GFP) and mononuclear phagocytes in green (tdTomato). Elapsed time is shown at the upper right. Fourteen stacks (depth: 53.5 μm) were recorded with 5 min intervals with a PlanApo 20x/0.8 objective with 0.7 zoom and exported to video at 9 fps using ImageJ (3D-Projection). The scale bar indicates 50 μm.

Supplementary Video 11 related to Figure 8 Bacterial clearance by neutrophils in close vicinity of CX_3_CR1 positive cells. UPEC strain is displayed in magenta (GFP), neutrophils in cyan (eFluor 660 conjugated anti-Ly6G mAb) and CX_3_CR1 positive cells in green (tdTomato). Elapsed time is shown at the upper left corner. Thirty five stacks (depth: 83.2 μm) were recorded with 5 min intervals with a PlanApo 20x/0.8 objective with 0.6 zoom and exported to video at 9 fps. The scale bar indicates 50 μm.

Supplementary Video 12 Live imaging with a WPlanApo 40x/1.0 objective with 0.5 zoom of the kidney of a CX_3_CR1^+/GFP^ mouse where one tubule was infused with PBS and one with the UPEC strain, recorded 3.5 h after infection. The i.v. injection of TRITC dextran 500 kDa as contrast agent allow for blood flow assessment as the circulating erythrocytes replace the dectran and appear as black (not stained) dots. Seconds 0:00 to 0:03 present the untouched surrounding area, 0:04 to 0:21 the UPEC infected tubule and 0:21 to 0:25 the PBS infused tubule. Sequences were recorded at a rate of 2 fps for 150 s and then exported to video at 12 fps using ImageJ.

Figure S1 Post-surgery pictures of the experimental setting taken before transferring the animal to the microscope **(A)** or before tubule micro infusion **(C)** and of the respective stabilizing window used **(B,D)**.

Figure S2 Confocal tile scan of a live kidney 60 min post laser-induced tissue damage. Exposing the kidney to a short (10 s) burst of high intensity 405 nm laser (50 mW) induced a small scope of approximately 0.1 mm^2^ in the renal cortex. Within 60 min the area surrounding the scope was infiltrated with Ly-6G positive neutrophils (magenta). Mononuclear phagocytes displayed in green (anti-F4/80-mAb), renal capillaries in red (anti-CD31-mAb) and neutrophils in magenta (anti-Ly6G-mAb). The scope is outlined by white arrows. The scale bar indicates 100 μm.
