# Supplementary material for: High Resolution Intravital Imaging of the Renal Immune Response to Injury and Infection in Mice
Source: Front Immunol. 2019 Nov 29;10:2744. doi: 10.3389/fimmu.2019.02744 (PMC6916672; doi:10.3389/fimmu.2019.02744)
Supplement: Supplementary file 1 [file Table_1.DOCX]

**Supplementary table1**

*Table 1. Antibodies used for in vivo staining*

| **Antigen** | **Clone** | **Fluorophore** | **Dose**  (µg/g body weight) | **Concentration** (mg/ml) | **Supplier** |
| --- | --- | --- | --- | --- | --- |
| F4/80 | BM8 | Alexa647 | 0.2 | 0.2 | BioLegend |
| F4/80 | BM8 | BV650 | 0.4 | 0.2 | BioLegend |
| CD31 | 390 | Alexa555 | -- | 0.5** | eBioscience* |
| CD31 | 390 | eFluor450 | 0.2 | 0.2 | eBioscience |
| Ly-6G (Gr-1) | RB6-8C5 | Alexa488 | 0.4 | 0.5 | BioLegend |
| Ly-6G (Gr-1) | RB6-8C5 | Alexa555 | -- | 0.5** | eBioscience* |
| Ly-6G (Gr-1) | RB6-8C5 | BV421 | 0.15 | 0.2 | BD Bioscience |
| Ly-6G (Gr-1) | RB6-8C5 | eFluor660 | 0.2 | 0.2 | eBioscience |
| CD41 | MWReg30 | BV605 | 0.2 | 0.2 | BioLegend |

*Conjugated with Molecular Probes® Antibody Labeling Kit
**Concentration of unconjugated antibody
