# Supplementary figures and images for: High Resolution Intravital Imaging of the Renal Immune Response to Injury and Infection in Mice
Source: Front Immunol. 2019 Nov 29;10:2744. doi: 10.3389/fimmu.2019.02744 (PMC6916672; doi:10.3389/fimmu.2019.02744)

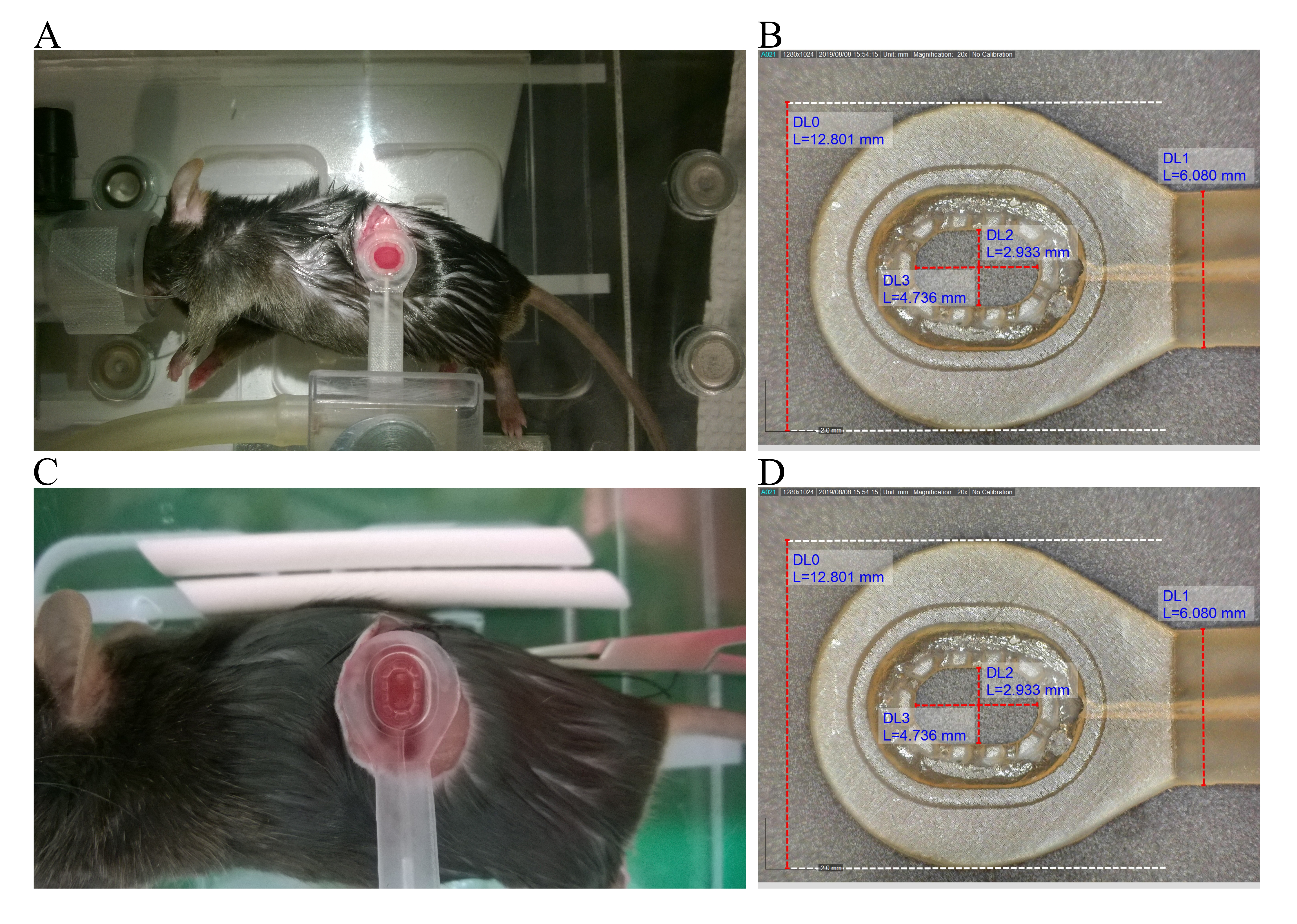

Supplement: Figure S1 — Post-surgery pictures of the experimental setting taken before transferring the animal to the microscope (A) or before tubule micro infusion (C) and of the respective stabilizing window used (B,D). [file Image_1.JPEG]

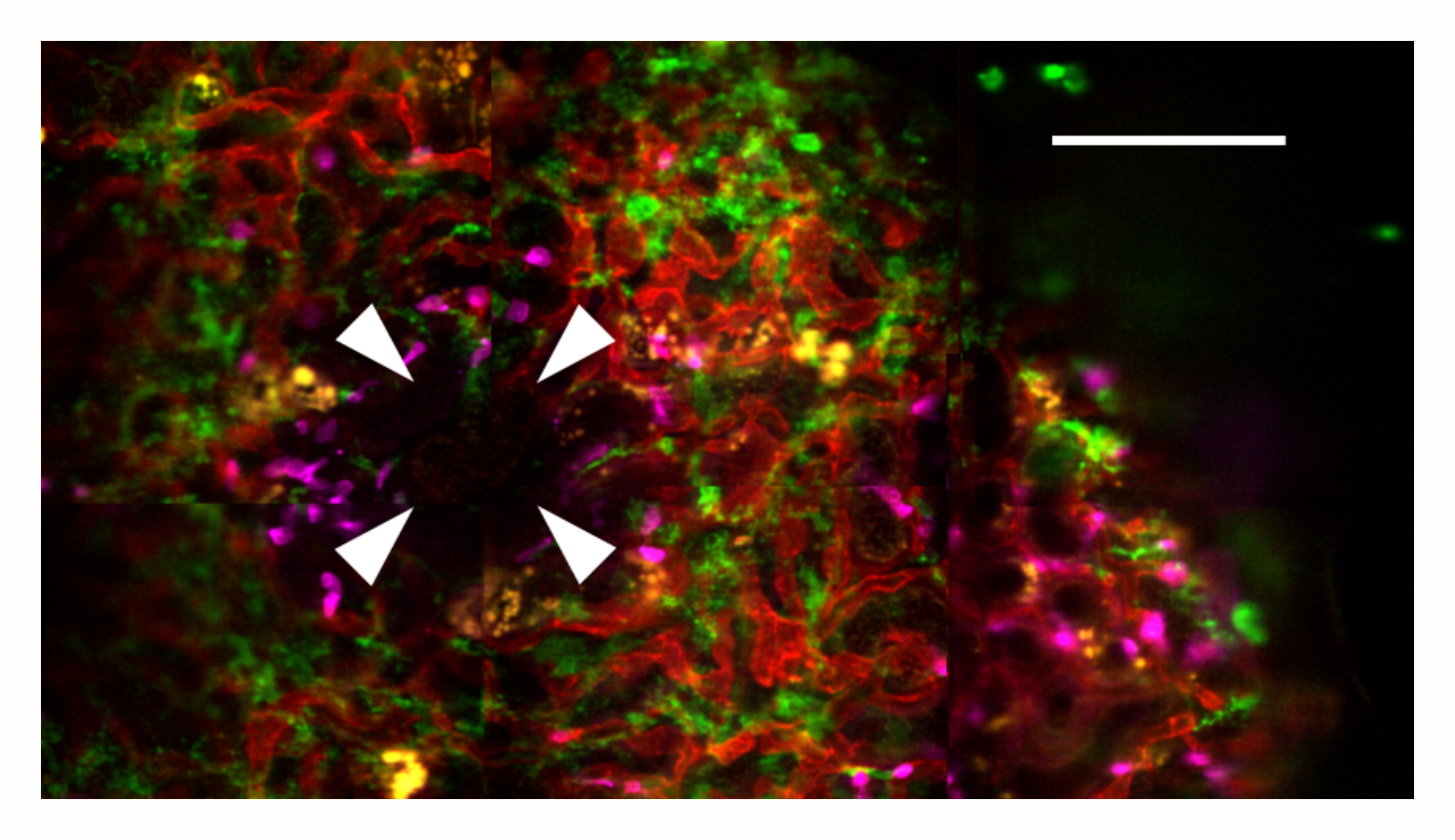

Supplement: Figure S2 — Confocal tile scan of a live kidney 60 min post laser-induced tissue damage. Exposing the kidney to a short (10 s) burst of high intensity 405 nm laser (50 mW) induced a small scope of approximately 0.1 mm2 in the renal cortex. Within 60 min the area surrounding the scope was infiltrated with Ly-6G positive neutrophils (magenta). Mononuclear phagocytes displayed in green (anti-F4/80-mAb), renal capillaries in red (anti-CD31-mAb) and neutrophils in magenta (anti-Ly6G-mAb). The scope is outlined by white arrows. The scale bar indicates 100 μm. [file Image_2.JPEG]
